# Ancestral dietary change alters the development of *Drosophila* larvae through MAPK signalling

**DOI:** 10.1080/19336934.2022.2088032

**Published:** 2022-06-29

**Authors:** Samuel G. Towarnicki, Neil A. Youngson, Susan M. Corley, Jus C. St. John, Richard G. Melvin, Nigel Turner, Margaret J. Morris, J. William O. Ballard

**Affiliations:** a School of Biotechnology and Biomolecular Sciences, The University of New South Wales, Sydney, NSW, Australia; b Department of Pharmacology, School of Medical Sciences, The University of New South Wales, Sydney, NSW, Australia; c The Institute of Hepatology, The Foundation for Liver Research, London, UK; d Adelaide Medical School, University of Adelaide, Adelaide, SA, Australia; e Department of Environment and Genetics, La Trobe University, Melbourne, VIC, Australia; f Department of Ecology, Environment and Evolution, School of Life Sciences, Victoria 3086, La Trobe University, Melbourne, VIC, Australia

**Keywords:** Diet, Nutrition, Epigenetics, Phenocopy

## Abstract

Studies in a broad range of animal species have revealed phenotypes that are caused by ancestral life experiences, including stress and diet. Ancestral dietary macronutrient composition and quantity (over- and under-nutrition) have been shown to alter descendent growth, metabolism and behaviour. Molecules have been identified in gametes that are changed by ancestral diet and are required for transgenerational effects. However, there is less understanding of the developmental pathways altered by inherited molecules during the period between fertilization and adulthood. To investigate this non-genetic inheritance, we exposed great grand-parental and grand-parental generations to defined protein to carbohydrate (P:C) dietary ratios. Descendent developmental timing was consistently faster in the period between the embryonic and pupal stages when ancestors had a higher P:C ratio diet. Transcriptional analysis revealed extensive and long-lasting changes to the MAPK signalling pathway, which controls growth rate through the regulation of ribosomal RNA transcription. Pharmacological inhibition of both MAPK and rRNA pathways recapitulated the ancestral diet-induced developmental changes. This work provides insight into non-genetic inheritance between fertilization and adulthood.

## 1. Introduction

Evidence accumulated across animal and plant research over the last 20 years has confirmed that the inherited determinants of an organism’s phenotype are more than just DNA [1]. Inheritance of RNA, protein and metabolites in the male or female gamete can influence various traits such as size, shape, behaviour and health [2,3]. Furthermore, ancestral environmental exposures can affect the levels and types of inherited molecules. The consequences of these molecules on descendent phenotype may range from short-term adaptation to a longer-term DNA mutation and selection [4,5], which can contribute to an individual’s disease risk [6].

Ancestral environmental changes that have been found to alter phenotypes of descendants include behavioural stress, toxin exposure and nutritional variation [1–3]. The latter is perhaps the most studied, and examples of ancestral diet altering descendent phenotype due to over- or under-nutrition have been documented in natural populations, including humans [7] and laboratory organisms such as *Caenorhabditis elegans* [8,9], *Drosophila* [10–14] and rodents [7]. In these studies, changes to descendent metabolism and growth often result in altered developmental timing, organ size, and body weight. For example, in *Drosophila*, paternal sugar exposure reprogrammed offspring lipid metabolism, leading to increased stored triglyceride levels [10]. Over 100 studies in rodents have also shown reprogramming of offspring feeding behaviour, birth and adult body weight, adiposity, insulin/glucose metabolism, hypertension etc., due to maternal or paternal obesity or starvation [7]. Another essential feature of developmental programming is that the effect on descendants is dependent on the time point of exposure to the environmental change [15]. Frequently, effects are noted in the offspring generation but are absent, weaker or even different in the grand-offspring generation [7]. This is due to offspring constantly experiencing the exposure directly, either across the placenta while *in utero* or as exposure to germ cells that will become offspring. The transmission mechanisms can also be indirect, for example, through faecal microbiota transfer, due to alterations in maternal care (including lactation). However, a major focus of the developmental programming field has been identifying molecules in the gametes that drive descendent phenotypes [2]. Epigenetic changes such as DNA methylation, histone modifications and non-coding RNAs have all been found to alter offspring gene expression resulting in developmental and adult phenotype differences. In the Drosophila system mentioned above [10], sugar exposure extensively altered repressive sperm histone methylation patterns in the exposed males, with fat metabolism genes being particularly affected and prone to subsequent transcriptional changes in offspring, but not grand offspring. In mammals, the physiological intimacy of gestation means that developmentally programmed offspring phenotypes are hard to prove due to gamete-derived molecules if the mother is the exposed parent. However, this is not the case when the father is the exposed parent, and there are strong examples of paternal diet- and stress-induced offspring phenotypes being due to alterations in sperm small non-coding RNAs [16–18].

In contrast to the abundance of studies that have searched for a causative epigenetic change in the gamete, there is less information on the post-fertilization mechanisms that bridge the reading of the inherited molecule and the ultimate cell, tissue or whole organism phenotypes. As mentioned, most developmental programming studies have reported metabolic phenotypes. Work by ourselves and others has suggested that Foxo1 mediates developmentally programmed changes to pancreas function in mammals- [19] and Myc-directed pathways [20]. However, less has been reported on changes to pathways that regulate offspring growth and developmental timing. Therefore, in this study, we aimed to understand the developmental pathways affected by ancestral diet at different offspring life stages.

**Figure 1.**
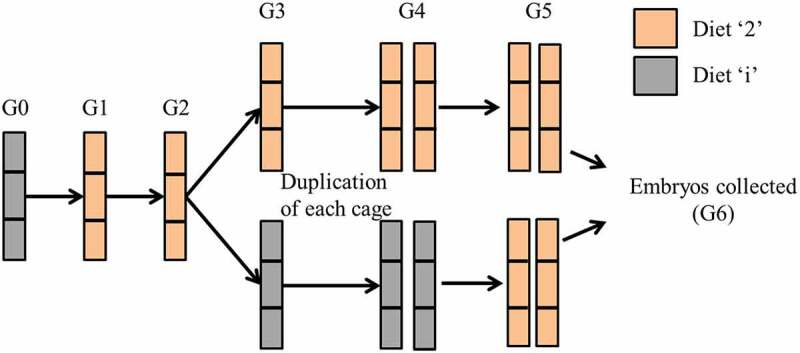
**Overview of experimental design**: The generational design allowed comparison of G6 embryos produced by great-grandparents and grandparents that differed in their dietary composition, with parents provided the same diet. Diets ‘4’ and ‘6’ were included as additional foods at G3, G4 and G5 only.

In our previous study, we observed generational differences in reproductive fitness between isosequential *Drosophila* strains harbouring distinct mitochondrial DNAs (mitotypes) that were fed diets of varying protein to carbohydrate (P:C) ratios [21]. Large shifts in relative proportions of mitotypes within-population cages were seen in generations 1 to 4 but not in later generations. These differences in comparative mitotype fitness were reversible with a diet switch indicating that genetic selection was not responsible. We hypothesized that the within-strain generational differences could be due to transgenerational epigenetic inheritance effects. Notably, in our and other studies, effects accumulated across generations suggest that the fly’s diet, when it produced its gametes, was not the sole cause of the developmental differences [12,13]. We, therefore, designed our experiments to exclude parental effects by ensuring that all parents consumed the same diet, and variation in ancestral diet was limited to the great grand-parental and grand-parental generations (Figure 1). Our studies suggest that ancestral P:C diet-induced descendent developmental timing changes are due to alteration of MAPK signalling pathways in the period between the embryo and pupal stages. While theoretically possible, we suggest that it is exceedingly unlikely that independent mutations arose in the same pathways in three separate experimental blocks (i.e. completely separate multi-generational experiments).

## 2. Materials and methods

(a) Diets and fly genotypes

We utilize four diets in this study that differ in their P:C ratios. Three of these diets, 1:2, 1:4 and 1:6 P:C (referred to as diet ‘2’, diet ‘4’, and diet ‘6’, respectively) are constructed diets, and one, Formula 4–24 instant *Drosophila* medium (Carolina biological supply company, referred to as diet ‘i’) is a pre-made diet. Diets 2, 4, and 6 were constructed as Aw *et al*. [21] described. We use the Alstonville; *w*^1118^ strain of flies used by Aw *et al*. [21], which were isosequential due to balancer chromosomes. The study of Aw *et al*. also confirmed that transgenerational effects of ancestral diet on *Drosophila* phenotype might vary with the genotype. When fed the 1:2 P:C diet in a population cage, we observed that the frequencies of these flies plateaued after generation 4 (Figure 1(a) [21]). As the four stains in the cage harboured distinct mitochondrial haplotypes but the same *w*^1118^ background, we were interested in exploring the possibility that this result was non-genetic in this follow-up study. The reproducibility of larval development including transgenerational effects has been established in a previous study. Aw *et al*. [21] reproduced the relative larval developmental time of the strains harbouring the Alstonville and Dahomey mitochondria first in an Oregon R background and second in a Canton S nuclear background.

To ensure the flies used in the current studies were isosequential and slightly deleterious mutations had not accumulated, three independently maintained populations of Alstonville, *w*^1118^ females, were each backcrossed to *w*^1118^/*w*^1118^ males for three generations before the generational experiment. The *w*^1118^/*w*^1118^ males were each sourced from three independent populations. White eye colour and mtDNA type were confirmed before and after each study to ensure no contamination with other fly strains in the laboratory [21]

(b) Generational experimental design

The experimental design differed from our previous study, which aimed to look at the frequency of flies in population cages [21]. We utilized a six-generation (G6) experimental design that controlled the number of generations in which flies were fed each diet (Figure 1). To determine the robustness of the results, we conducted three non-overlapping experimental blocks where the founding females for each block were derived from a different set of backcrossed lines. We then tested experimental replicates within blocks.

A single population cage was initially established with ~800 adult Alstonville flies fed diet ‘i’ (termed generation 0). Oviposition medium consisting of 10% treacle and 4% agar with a thin spread of baker’s yeast was placed into the cage in a small petri dish (3 cm in diameter). This medium was replaced daily for 3 d. On the third day, flies were provided with the oviposition medium for 6 h before removing the medium containing embryos. Embryos were collected from the agar plate onto a fine mesh screen using a water stream from a wash bottle. They were then washed with 4% bleach to remove microbes, thoroughly rinsed with distilled water, and transferred to 60 × 130 mm bottles containing food [22]. Embryos were placed into 12 bottles containing diet ‘2’ (generation 1) at ~200 embryos in each bottle, with four bottles per population cage. In each generation, four adult stock Alstonville and four adult stock Dahomey [21] males maintained on diet ‘i’ were ground in 1400 µl of ddH_2_O, and 140 µl of the solution was added to each bottle to standardize the microbiome. We did not evaluate microbial composition in this study. Aw et al. [21] found that dietary change from a 1:2 P:C diet to a 1:16 P:C diet does not significantly change the relative microbiota composition in Alstonville flies.

For all generations, day 0 was determined to be when the first flies eclosed. Flies from that day were discarded. Bottles were opened, and adult flies were allowed to populate the cages for 72 h. The bottles were then removed. Oviposition medium was placed in a small petri dish, embryos were collected as described previously, and established on the diet ‘2’ again (generation 2).

Embryos collected from generation 2 were used to seed bottles with diets ‘i’, ‘2’, ‘4’ or ‘6’ (generation 3). Embryos from generation 3 were moved onto the same diet as their parents (i.e. ‘i’ to ‘i’, ‘2’ to ‘2’ etc.; generation 4), and the number of cages was doubled at this generation from 3 to 6 cages. Finally, embryos from generation 4 were placed onto bottles containing diet ‘2’ (generation 5).

**Figure 2.**
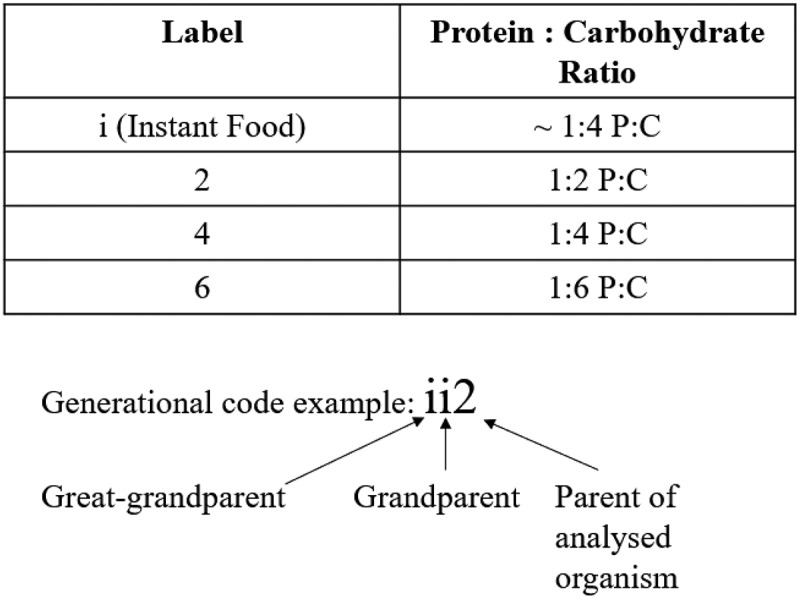
**Diet designations**: Each analysed organism is referred to by the diet treatment fed to the preceding three generations. The example refers to an organism whose: great grandparents ate the ‘i’ diet, grandparents ate the ‘i’ diet, and parents ate the ‘2’ diet, resulting in a label of ‘ii2’.

Embryos produced by generation 5 flies were collected within 45 minutes of laying to ensure single-celled zygotes were used for RNA-seq, RT-qPCR, and development assays. Collected embryos (generation 6) are referred to as their previous three ancestral generations. Those maintained consistently on diet ‘2’ are termed 222, while those fed different ancestral diets are termed ii2, 442, and 662 (Figure 2). We consider the ii2 diet to be the control diet.

(c) Development time and adult weight

Development time of generation 6 embryos was conducted using diet ‘2’. It was measured as a count of adult flies eclosing (hatching) in 3d and the time to pupariation of G6 flies and G5 flies. These development time assays were conducted on three biological blocks and provided three independent measures. Pupariation time was also independently measured for larvae fed diets with the MAPK and ribosome inhibitors. Time to pupariation is calculated as the time in hours from the midpoint of the egg-laying period plus larval development time. Ten embryos per vial of the experimental diet were established with ten treatment blocks. Pupae were individually time-stamped on the side of the vial every 6 hours during daylight hours, and the mean development time of each vial was calculated. Pupariation was identified by observation of everted pupal spiracles.

For the eclosion studies, ten replicate bottles containing approximately 200 embryos were prepared for each diet as described for the generational experiment. Adult flies were cleared from bottles on the first day that eclosion was observed. Flies were then allowed to eclose for 72 h, after which they were collected and counted as the 3d eclosion period.

Adult weight of generation 6 flies was collected from development assays. Flies were initially weighed for wet weight, then incubated at 55°C for 72 h and weighed again for dry weight. Ten groups of five females were measured for each treatment in two independent blocks.

(d) Transcriptome analysis of embryos

Generation 6 embryos were snap-frozen in liquid nitrogen. RNA was isolated from ~20 embryos per cage using the PicoPure RNA isolation kit (Arcturus). DNAse treatment was performed using the RNase-free DNase set (Qiagen). RNAseq was performed at the Ramaciotti Centre for genomics (UNSW). RNA was extracted from five biological replicates of each 222 and ii2 diet group (10 samples in total) using the TruSeq Stranded mRNA-seq kit (Illumina, DA, USA). The samples were sequenced in one flowcell on the NextSeq 500, producing paired-end reads of 75bp.

Reads from the ten samples were mapped to the NCBI *D. melanogaster* genome GCF_000001215.4_Release_6_plus_ISO1_MT using the Subread package (v 1.6.3) [23]. Mapped reads were assigned to features using the featureCounts function of the Subread package, with an average 33.5 M reads assigned per sample. Lowly expressed genes (cpm < 0.5 in at least four samples) were filtered out. Differential expression analysis was performed using the R environment (R v3.6.3) and the Bioconductor packages edgeR v3.28.1 [24] and limma (voom) v3.42.2 [25]. A DGEList object was created using edgeR, and the data were normalized using the TMM method. This DGEList object was used as input in the voom analysis. The count data were log-transformed before applying the lmFit and eBayes functions to test for differential expression. The commands used to carry out the D.E. analysis are included in Supplementary DE_analysis.txt. Differential expression analysis with edgeR and limma (voom) produced 1550 and 1403 differentially expressed genes (adjusted p.value (Benjamini Hochberg) < 0.05). As 98% of the voom DEGs were also detected by edgeR we used this robust set of genes for downstream analysis. Functional analysis was performed using limma’s kegga and goana functions, the clustering functions within the STRINGdb package v1.26.0 [26] and the enricher function in the clusterProfiler package v3.8 [27].

(e) Pathway validation

Expression of genes of interest was performed using RT-qPCR from 5 replicates/ gene from each of 2 independent blocks (10 total) (Table S1). RNA was isolated using Trizol reagent, and cDNA was prepared using Superscript II RNase (ThermoFisher). Following Aw et al. [21], the expression of genes of interest was calculated relative to the average expression of housekeeping genes using the comparative cycle threshold (Ct) method. The efficiency of amplification was above 95%.

(f) Phenocopy of ancestral-diet induced effects on developmental timing with pharmacological inhibition of MAPK and ribosome biogenesis pathways.

Chemical inhibition of developmental pathways can lead to morphological differences resulting in a copy of a phenotype (phenocopy) distinct from their normal phenotype [21]. Chemical inhibitors can also provide partial inhibition, advantageous over knockouts when complete inhibition is lethal. In *Drosophila*, progression to the next larval stage is triggered by the larva attaining the necessary body weight. Reaching critical body weight activates a MAPK signalling cascade and upregulates ecdysone biosynthetic genes [28,29]. Furthermore, altered MAPK signalling may affect ribosomal RNA biogenesis and cellular growth [30].

To phenocopy the slower rate of development of flies fed the ii2 diet, we partially inhibited the MAPK and ribosome biogenesis pathways of the flies fed the 222 diet. Preliminary titrations showed that for the MAPK pathway, *Egfr* was inhibited with 100 μM Tyrphostin AG1478, and *rolled* was inhibited using 2.5 μM SCH772984. Ribosome biogenesis was inhibited at *pol1* using 50 μM BMH-21. Each compound was administered in individual experiments. Development time and gene expression were then analysed.

(g) Statistical analyses

All data were analysed using JMP Pro 16.0.0 (SAS Institute 2021). The interquartile range (IQR) was calculated for all values from the same treatment group (IQR = Q3 – Q1, where Q3 and Q1 are the third and first quartiles of the fitted standard normal distribution). We considered data points smaller than Q1 – 1.5 IQR or greater than Q3 + 1.5 IQR as outliers, and they were removed. For development time, we include the mean development time of each vial. One-way ANOVAs investigated the effect of ancestral diet on time to pupation and 3d eclosion. Two-way ANOVAs of the log-transformed ΔCt data compared the expression of differentially expressed genes whose ancestors were raised on the ii2 and the 222 diets. The primary assumption of the pairwise approach is that the additive effect of concentration, gene, and replicate can be adjusted by subtracting the Ct number of the target gene from that of the reference gene [31]. Data from the distinct studies were combined as no significant block effects were detected. Differences in developmental time and expression were explored *Post-hoc* using two-tailed Student’s t-tests [21].

## 3. Results

(a) Development time and adult dry weight

**Figure 3.**
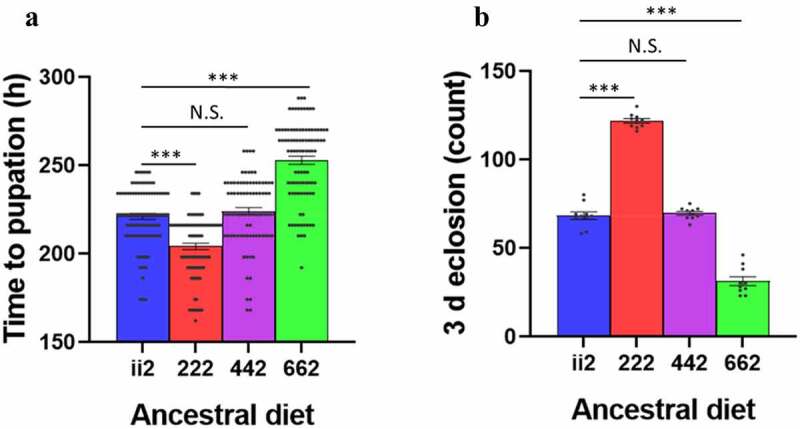
**Development assays**: Time to pupation (**a**) and 3 d eclosion (**b**) of the 222 flies was faster than that of the ii2 and 442 flies, while the 662 flies were the slowest to develop. Bars indicate mean values ± SEM. Post-hoc Student’s t-tests compared ii2 to all other diets in each assay. N.S. indicates not significant, *** indicates p < 0.001.

Flies raised on the 222 dietary regimes developed significantly faster than all other treatments, with the ii2 and 442 regimes having the same rate of development and the 662 set having the slowest (Figure 3(a, b)). ANOVAs showed that ancestral diet significantly affected time to pupation (F_3, 27.1_ = 87.4, p < 0.0001) and 3 d eclosion (F_3, 36_ = 225.69, p < 0.0001). The effects on developmental timing were also seen when a single previous generation was exposed to a particular diet (Fig. S1). This indicated that a single generation could induce the ancestral diet effect rather than accumulative or exacerbated by an increased number of exposures.

Adult dry-weight was measured to determine if the faster development of the 222 flies was perhaps a trade-off for lower body size, which is correlated with adult fecundity [32]. A Student’s t-test between ii2 and 222 flies showed no significant difference in adult female body weights (t_18_ = 0.17, p = 0.87).

(b) Transcriptome analysis of embryos

**Figure 4.**
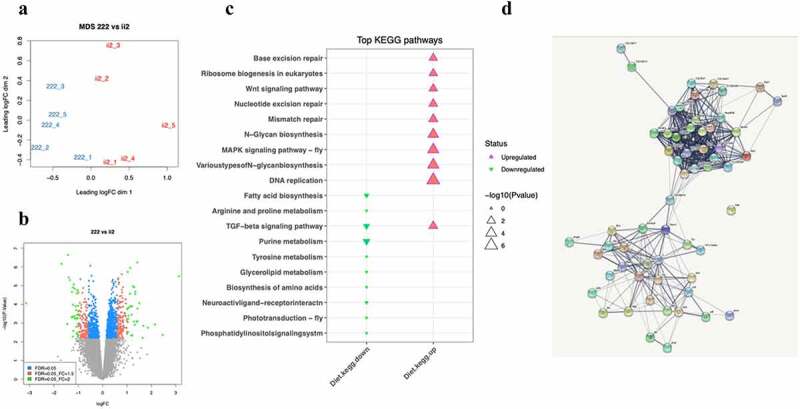
**RNAseq analysis**: (**a**) Multidimensional scaling (MDS) plot showing separation between samples (222 = 3 generations fed on 1:2 Protein: Carbohydrate diet, ii2 = change in ancestral diet). (**b**) Volcano plot showing gene expression changes associated with the 222 diet. (**c**) Top KEGG pathways as found using kegga (limma). (**d**) Genes involved in the Ribosome and MAPK signalling pathways had a fold change between the 222 diet and the ii2 diet with an FDR < 0.1. Edges indicate an interaction between genes.

The ancestral diet-induced differences in developmental timing were evident in the period between the embryonic and pupal stages. Therefore, we performed RNA-seq on ii2 and 222 embryos to identify formative transcriptional signatures that could drive the differences in growth timing. A multidimensional scaling plot demonstrated that the five replicates of the 222 flies separated from the five replicates of the ii2 flies along with the main principal component consistent with a different transcriptional signature in these conditions (Figure 4(a)). Differential expression analysis with limma (voom) produced 1403 differentially expressed genes (DEGs) (adjusted p.value < 0.05). Eight hundred fifty-one of the voom DEGs were upregulated, and 552 were downregulated in the 222 samples compared to ii2, as illustrated by the volcano plot (Figure 4(b)). Pathway and network analysis of the DEGs revealed that growth-associated pathways such as ribosome biogenesis, MAPK signalling and Wnt signalling were higher in 222 compared to ii2. The top upregulated and downregulated KEGG pathways found using the kegga (limma package) are shown (Figure 4(c)). The KEGG pathway results are included in Supplementary Table S2. The interaction network between the genes in the Ribosome and MAPK signalling pathways was determined using STRINGdb (Figure 4(d)).

(c) Pathway validation

**Figure 5.**
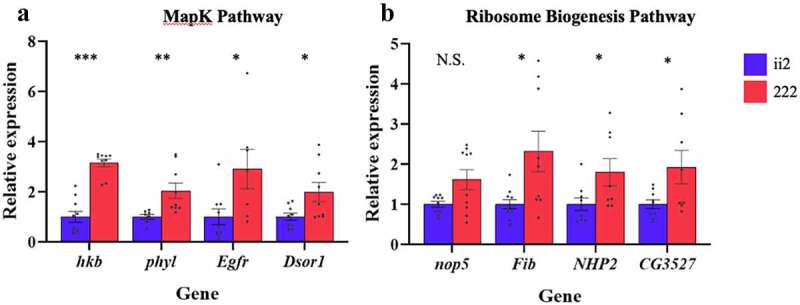
**Pathway validation**: Expression of MAPK signalling pathway genes (**a**) and Ribosome biogenesis genes (**b**) was higher for the 222 flies. Bars indicate relative expression ± SEM. N.S. indicates not significant * indicates p < 0.05, ** indicates p < 0.01, *** indicates p < 0.001.

We focused our subsequent studies on the MAPK and Ribosome Biogenesis development pathways. Diet plays an essential part in activating MAPK pathways [33]. Aldridge and Maggert [34] reported that ribosomal DNA (rDNA) copy number polymorphisms can be created by manipulating the diet of flies. To validate the RNAseq results, we compared the expression of four differentially expressed genes from the MAPK and Ribosome Biogenesis development pathways from embryos whose ancestors were raised on the ii2 and the 222 diets (Figure 5).

Two-way ANOVA comparing the expression of four MAPK genes *hkb, phyl, Egfr*, and *Dsor1* genes in larvae fed two ancestral diets shows a significant effect of diet (F_1, 65_ = 22.52, p < 0.0001) but no significant effect of gene (F_3, 65_ = 0.24, p = 0.087) or a gene by diet interaction (F_3, 65_ = 0.37, p = 0.78). To further investigate these results, we conducted post hoc t-tests investigating the influence of ancestral diet on expression diet. The MAPK genes *hkb, phyl, Egfr*, and *Dsor1* all showed significant effect of ancestral diet (t_17_ = 5.60, p < 0.0001; t_15_ = 3.72, p = 0.002, t_14_ = 2.68, p = 0.02; t_16_ = 2.63, p = 0.02, respectively).

Two-way ANOVA comparing the Ribosome Biogenesis genes *nop5, Fib, NHP2*, and *CG3527* and two ancestral diets shows a significant effect of diet (F_3, 65_ = 22.52, p < 0.0001) no significant effect of gene (F_3, 65_ = 0.24, p = 0.87) or a diet by gene interaction (F_3, 65_ = 0.37, p = 0.78). Expression of three of the Ribosome Biogenesis genes: *Fib, NHP2*, and *CG3527* were significantly affected by ancestral diet (t_17_ = 2.79, p = 0.01, t_15_ = 2.42, p = 0.03; t_15_ = 2.30, p = 0.04, respectively). The expression of *nop5* was not significantly affected by diet (t_18_ = 1.90, p = 0.074).

(d) Phenocopy of ancestral diet induced effects on developmental timing with pharmacological inhibition of MAPK and ribosome biogenesis pathways

We were able to phenocopy the ancestral diet-induced effects on developmental timing through pharmacological inhibition of both MAPK proteins ERK1/2 and EGFR as well as ribosomal biogenesis (Fig. S2). These experiments confirmed the hierarchy of the two processes as inhibition of MAPK proteins affected rRNA transcript levels but not *vice versa.*

**Figure 6.**
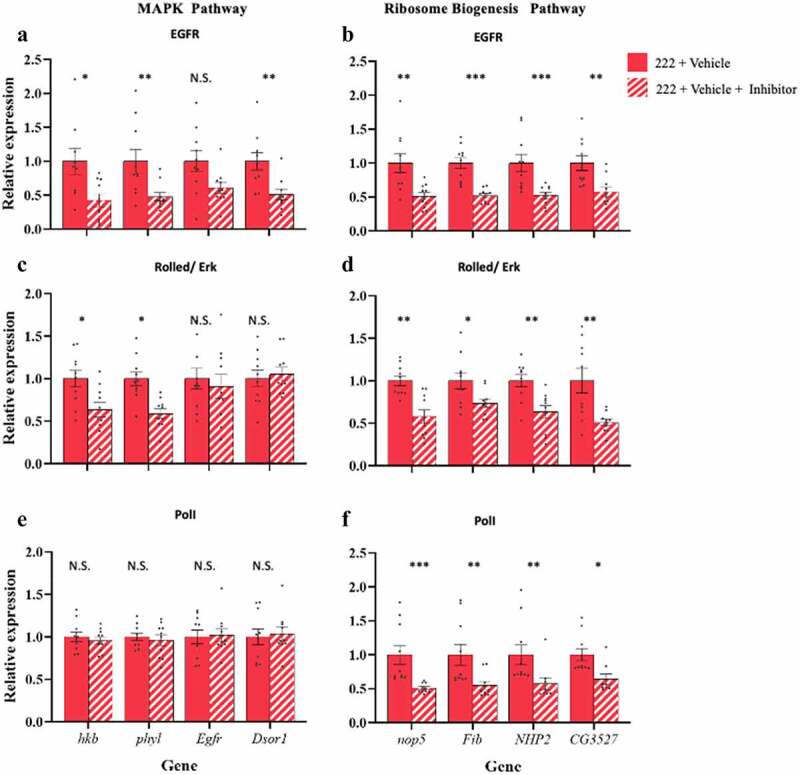
**Phenocopy of ancestral diet induced transcriptional changes with pharmacological inhibition of target pathways**: Blocking of MAPK with 100 μM Tyrphostin AG1478 (EGFR inhibitor, A, B) or 2.5 μM of SCH772984 (Rolled/Erk inhibitor, C, D), showed a reduction of MAPK gene expression at the point of inhibition and downstream (**a**, **c**) but also led to a reduction in expression of ribosome biogenesis pathway genes (**b**, **d**). Partially inhibiting ribosome biogenesis with 50 μM of BMH-21 (Pol 1 inhibitor, E, F) showed no change in MAPK expression (**e**). There was, however, a reduction in the expression of ribosome biogenesis pathway genes (**f**). Bars indicate relative expression ± SEM. Post-hoc Student’s t-tests N.S. indicates not significant, * indicates p < 0.05, ** indicates p < 0.01, *** indicates p < 0.001.

Partially blocking MAPK at EGFR with 100 μM Tyrphostin AG1478 in 222 flies showed reduced expression of seven of eight genes tested (Figure 6(a, b)). For the MAPK pathway ANOVA showed a significant effect of treatment (F_1, 71_ = 28.45, p < 0.0001), gene, (F_3, 71_ = 0.88, P = 0.46) and a treatment x gene interaction (F_3, 71_ = 0.85, P = 0.47). Post hoc t-tests showed significant effect of treatment for *hkb, phyl*, and *Dsor1* (t_17_ = 2.83, p = 0.01; t_18_ = 3.20, p = 0.005; t_18_ = 3.67, p = 0.002, respectively), but treatment was not significant for *Egfr* (t_18_ = 1.5719, p = 0.13; Figure 6(a)). For the Ribosome biogenesis pathways ANOVA showed a significant effect of treatment (F_1, 72_ = 66.67, p < 0.0001) but no significant effect of gene or the interaction (F_3, 72_ = 0.22, p = 0.88, F_3, 72_ = 0.087, p = 0.97, respectively). Post hoc t-tests showed significant effects of treatment for *nop5, Fib, NHP2*, and *CG3527* (t_18_ = 3.64, p = 0.002; t_18_ = 5.96, p < 0.001; t_18_ = 3.96, p = 0.0009; t_18_ = 3.64, p = 0.002, respectively; Figure 6(b)).

Partial blocking of MAPK at ERK1/2 with 2.5 μM SCH772984 in 222 flies showed reduced expression of MAPK genes and the Ribosome biogenesis pathways downstream of the inhibitor (Figure 6(c,d)). ANOVA showed a significant effect of treatment (F_1, 72_ = 7.49, p = 0.01), but no significant effect of gene or the interaction (F_3, 72_ = 2.06, P = 0.11, F_3, 72_ = 2.19, P = 0.10, respectively). Post-hoc t tests showed significant effect of treatment for *hkb* and *phyl*, (t_18_ = 2.56, p = 0.02, t_18_ = 2.67, p = 0.02, respectively, Figure 6(c)), with no difference in expression upstream (t_18_ = 0.70, p = 0.49, t_18_ = 0.60, p = 0.56 for *Egfr*, and *Dsor1*, respectively, Figure 6(c)). As expected, Ribosome biogenesis pathways were down-regulated. ANOVA showed a significant effect of treatment (F_1, 72_ = 41.14, p < 0.0001), but no significant effect of gene or the interaction (F_3, 72_ = 1.42, p = 0.24, F_3, 72_ = 0.95, p = 0.42, respectively). Post-hoc t tests showed a significant effect of treatment for *nop5, Fib, NHP2*, and *CG3527* (t_18_ = 3.98, p = 0.001, t_18_ = 2.48, p = 0.02, t_18_ = 3.06, p = 0.007, t_18_ = 3.26, p = 0.004, respectively; Figure 6(d)).

Partial inhibition of ribosome biogenesis with 50 μM of BMH-21 showed no inhibition of MAPK expression but reduced expression of ribosome biogenesis pathway genes (Figure 6(e,f)). There was no significant inhibition of MAPK pathway genes. ANOVA showed no significant effect of treatment, gene, or the interaction (F _1, 72_ = 0.001, p = 0.97, F_3, 72_ = 0.05, p = 0.98, F_3, 72_ = 0.29, P = 0.83, respectively). Post-hoc t tests showed no significant effect of treatment for *hkb, phyl, Egfr*, and *Dsor1* (t_18_ = 0.56, p = 0.59; t_18_ = 0.65, p = 0.52; t_18_ = 0.30, p = 0.77; t_18_ = 0.44, p = 0.66, respectively, Figure 6(e)). There was reduced expression of ribosome biogenesis pathway genes. ANOVA showed a significant effect of treatment (F_1, 70_ = 43.17, p < 0.0001), but no significant effect of gene or the interaction (F_3, 70_ = 0.97, p = 0.41, F_3, 70_ = 0.44, p = 0.73, respectively. Post-hoc t tests showed a significant effect of treatment for *nop5, Fib, NHP2*, and *CG3527* (t_18_ = 4.46, p = 0.0003; t_18_ = 3.29, p = 0.004; t_17_ = 3.15, p = 0.006; t_17_ = 2.41, p = 0.03, respectively; Figure 6(f)).

## 4. Discussion

Here we have shown in a *Drosophila* model that the MAPK pathway is a mediator of grand-parental dietary effects on prepupal developmental timing. The overall impact of a higher ancestral P:C ratio (e.g. P:C 1:2) is faster development through the embryo to pupal stages than a lower ratio (e.g. P:C 1:6). This result was highly reproducible, seen in three independent experimental blocks, and induced when the grand-parental, or the grand-parental plus great grand-parental generation had the diets.

The study design differed from our previous investigations of multiple generational dietary exposures [21]. The nutritional formulations and use of the Alstonville strain were the same. Still, there were crucial differences, such as the number of generations fed a particular diet and the number of fly strains used. It was essential to change the study design to remove the influence of parental diet on offspring development which has been well described [12–14,35–38]. In comparison with our previous work, the close similarity of the effects of a two-generation and one-generation dietary exposure (Fig. S1) suggests that the new model is not reproducing the accumulative multiple generational effects [21]. This may be due to the cumulative impact occurring after more generations (it was previously seen after four generations on a diet). Otherwise, it could be linked to inter-strain competition, as previously seen in flies kept in cages containing 2–4 different *Drosophila* strains.

In invertebrates, dietary protein levels have been found to contribute to growth, fecundity and ageing [4,39,40]. Faster developmental timing is observed when a high P:C ratio is consumed by larvae [21], by only the parental generation [37] and now, in this study, only in the grand-parental generation. A recent study of the effects of an ancestral ‘rich’ relatively high protein vs ‘poor’ lower protein diet also observed a faster developmental timing in the grand-offspring of the ‘rich’ diet flies [41]. Interestingly, they observed a greater influence of grand-parental diet than parental diet on various offspring phenotypes, including larval to pupal developmental timing. However, the study did not investigate the underlying growth regulatory pathways affected by the different generational dietary exposures diet.

Diet is a strong determinant of developmental timing in animals [42,43]. In *Drosophila*, the effects of varying carbohydrates, proteins and lipids on the timing of the various developmental stages have been examined, and the importance of the insulin/TOR pathway for regulation has been highlighted [42]. In this pathway circulating levels of amino acids, carbohydrates and *Drosophila* insulin-like peptides are detected by cell-surface receptors, which activate intracellular networks of signalling molecules including PI3K/AKT, FOXO and TOR, which in turn regulate processes required for growth such as transcription, protein synthesis and autophagy [42]. However, it is essential to note that in our study, the developmental timing changes in offspring were due to their ancestors, not their diet. Thus, the flies on the ii2 or 222 diets are expected to have qualitatively equivalent circulating levels of amino acids and carbohydrates. Indeed, the RNA-seq analysis did not indicate that this crucial growth regulatory pathway is responsible for the observed ancestral diet effects.

While providing new insight into how transgenerational effects are mediated in the post-fertilization period, our study does not provide evidence on the molecular change in the gametes that drives the subsequent developmental changes. A previous transgenerational study in *Drosophila* found rDNA copy number reduction in offspring of flies that consumed a high protein diet [34]. We found no evidence for this genetic change with qPCR for rDNA copy number (t_22_ = 0.45, p = 0.67, ii2 vs 222 larvae) in our study, which points to an epigenetic source of the growth phenotype. Mechanistic studies of the genes identified here could link the observed phenotypes to an epigenetic change in the gametes. The prominence of the MAPK pathway in our study’s network analysis of embryonic transcriptomes makes it tempting to predict that the transgenerational phenotypes are caused by the inheritance of histone modifications or small non-coding RNA that modulates this pathway. Future sophisticated cell- and stage-specific gene deletions of MAPK genes are needed to dissect the mechanisms involved.

Corroboration of the results we observe in a second and third genetic background is required to test the generality of our results. Strain genetics influence various diet responses, including metabolism, offspring development time and transgenerational phenotypes [44–46]. The *white* mutation in *Drosophila* has received particular attention as it is now known to influence a wide variety of metabolic pathways [44,47]. However, we and others have abundant evidence that transgenerational effects of diet can be strong determinants of phenotype regardless of the presence [10,11,21,48–50] or absence of the *white* gene mutation [14,21]. Indeed, there are parallels between the *Drosophila* white mutation and the mouse *agouti* gene variants in that mutations initially selected as tools for genetics research have uncovered epigenetic inheritance mechanisms and have profound effects on organismal metabolism [51,52]. Nonetheless, it is unclear whether the non-genetic effects we have observed here result from diet alone or the interaction of diet with the *white* genetic background. Future work on this model will determine the genetic influences on this novel transgenerational effect.

## Supplementary Material

Supplemental Material

## Data Availability

The data supporting this study’s findings are available upon reasonable request from the corresponding author, JWOB.

## Summary statement

Diet-induced changes in the timing of embryo to pupa development of descendant lineages are caused by alterations to MAPK signalling in *Drosophila*.
